# Licochalcone A: a review of its pharmacology activities and molecular mechanisms

**DOI:** 10.3389/fphar.2024.1453426

**Published:** 2024-08-12

**Authors:** Meihua Liu, Yang Du, Dejiang Gao

**Affiliations:** ^1^ Research Center of Emotional Diseases, Shenyang Anning Hospital, Shenyang, China; ^2^ Shenyang Key Laboratory for Causes and Drug Discovery of Chronic, Shenyang, China; ^3^ The First Affiliated Hospital of Dalian Medical University, Dalian, China

**Keywords:** Licochalcone A, licorice, anti-cancer, anti-inflammatory, targets

## Abstract

Licorice, derived from the root of Glycyrrhiza uralensis Fisch, is a key Traditional Chinese Medicine known for its detoxifying, spleen-nourishing, and qi-replenishing properties. Licochalcone A (Lico A), a significant component of licorice, has garnered interest due to its molecular versatility and receptor-binding affinity. This review explores the specific roles of Lico A in various diseases, providing new insights into its characteristics and guiding the rational use of licorice. Comprehensive literature searches using terms such as “licorice application” and “pharmacological activity of Lico A” were conducted across databases including CNKI, PubMed, and Google Scholar to gather relevant studies on Lico A’s pharmacological activities and mechanisms. Lico A, a representative chalcone in licorice, targets specific mechanisms in anti-cancer and anti-inflammatory activities. It also plays a role in post-transcriptional regulation. This review delineates the similarities and differences in the anti-cancer and anti-inflammatory mechanisms of Lico A, concluding that its effects on non-coding RNA through post-transcriptional mechanisms deserve further exploration.

## 1 Introduction

The root of *G. inflata* Batal has been a valuable medicinal resource for licorice widely used in Asia and worldwide. Licocalcone A (Lico A) is one of the characteristic component of the root of *Glycyrrhiza inflata*. The application of licorice dates back to ancient civilizations such as Greece and Rome (Armanini et al., 2002). Today, licorice is widely incorporated into food, medicinal products, health supplements, and cosmetics, recognized for its safety and efficacy. In Traditional Chinese Medicine, licorice is prized for its harmonizing properties, and it has also become popular in dietary applications for its health benefits (Herrera et al., 2009). Modern applications extend to food additives, tobacco flavoring, and skin depigmentation products (Rizzato et al., 2017). Its safety has been affirmed by the U.S. Flavor and Extract Manufacturers Association (Pastorino et al., 2018), solidifying its reputable status and prompting increased research into its pharmacological activities and applications (Pastorino et al., 2018).

**FIGURE 1:**
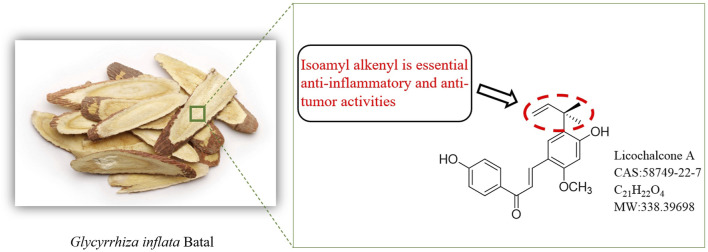
Origin and structure of Licochalcone A.

Flavonoids, common in nature (Perezvizcaino and Fraga, 2018), typically form glycosides in plants or exist in their free form (Vukics and Guttman, 2008). This structural diversity translates to varied pharmacological activities, including free radical scavenging, especially in flavonoids with catechol structures (Yang et al., 2000; Mukne et al., 2011; Cheng et al., 2019). Chalcones, a specific class of flavonoids, have a 1,3-diphenylpropenone skeleton. Among these, Lico A (Figure 1) stands out with its distinct structure and potent anti-inflammatory potential (Cui et al., 2008; Siddiqui et al., 2011; Kumar et al., 2014; Silva et al., 2018; Zhang et al., 2019).

Inflammation, a complex defense response to tissue injury, involves the vascular system and is triggered by inflammatory cytokines and mediators (Gabriele and Pucci, 2017; Huang et al., 2017). Studies show that Lico A mitigates LPS-induced effects by inhibiting inflammatory cytokine production and NO through NF-κB pathway suppression. Additionally, Lico A enhances the activity of antioxidant enzymes and protects against oxidative damage and cell death via ERK and Akt pathways (Hu and Liu, 2016). Furthermore, Lico A exhibits significant anti-tumor effects (Kang et al., 2017; Wu et al., 2017; Chen et al., 2018a), including the induction of apoptosis in cancer cells, regulation of the cell cycle, inhibition of tumor invasion and metastasis, and suppression of tumor angiogenesis (Hao et al., 2015; Lv et al., 2015).

Beyond its anti-inflammatory and anti-tumor properties, Lico A also demonstrates bacteriostatic, anti-parasitic, and osteogenic activities. This review summarizes the pharmacological actions and mechanisms of Lico A over the past five years, aiming to deepen the understanding of its bioavailability and inform further research.

## 2 Lico A exerts anticancer activity

Traditional Chinese herbal medicine, with its extensive history in treating tumors, continues to be a significant source of anti-tumor medications. Zhou et al. (2019). For example, Lico A is renowned for its potent anti-tumor activity (Daniell et al., 2000; Chen et al., 2017). Lico A’s anticancer effects manifest through various mechanisms, including inducing apoptosis in tumor cells, regulating the cell cycle to inhibit proliferation, curtailing tumor invasion and metastasis, and suppressing tumor angiogenesis by modulating related protein expression and signaling pathways (Yang et al., 2014; Kim et al., 2015; Park et al., 2015; Tsai et al., 2015; Yang et al., 2016; Kojima et al., 2017). Recent studies have highlighted Lico A’s cytostatic effects on human nasopharyngeal carcinoma cells mediated through apoptosis targeting the JNK/p38 pathway (Chuang et al., 2019).

Invasion and metastasis, key traits of malignant tumors, involve tumor cells detaching from the primary lesion, invading surrounding tissues or distant organs, and proliferating to form metastases. Lico A acts to inhibit this process by restraining cell migration, modulating E-cadherin and vimentin expression, and blocking MAPK and AKT signaling pathways (Mazzucchelli and Brambilla, 2000; Huang et al., 2019a). This multifaceted approach significantly reduces migratory and invasive capabilities of cells such as SCC-25. Additionally, tumor blood vessels, vital for supplying oxygen, nutrients, and growth factors, are also targeted by Lico A. Studies by Kim et al. (2010) demonstrated Lico A’s ability to inhibit neovascularization both *in vitro* and *in vivo* by suppressing angiogenesis factors such as IL-6, IL-8, and the VEGFR-2 signaling pathway. The effect of Lico A on different tumors and the specific mechanism of action is shown in Table 1.

**TABLE 1:** The type and mechanism of action of Lico A against cancer.

Type	Cell line	Dosage (μM)	Mechanism	Ref
Nasopharyngeal carcinoma	HONE-1, NPC-39, and NPC-BM	20–80	Target the JNK/p38 pathway and exerts a cytostatic effect	Chuang et al. (2019)
Breast Cancer	MDA-MB-231	10–100	Cell migration and invasion are inhibited by MAPK and AKT signaling	Huang et al. (2019b)
	MCF-7	20–100	Activate the LC3-II signaling pathway while suppressing the PI3K/Akt/mTOR/signaling pathway	Xue et al. (2017)
	3T3/MCF-7	IC_50_ = 27.57	Endogenous pathway-mediated apoptosis	Bortolotto et al. (2017)
	MCF-7 and MDA-MB-231	10–30	passed intracellular mitochondrial apoptosis pathway	Kang et al. (2017)
Glioma	M059K/U-251 MG/GBM8901	20–30	Induced ADAM9 expression and inhibits cell invasion activity through the MEK-ERK signaling pathway	Huang et al. (2018)
	U87	20–40	Inhibit the growth of cells by inducing cell cycle arrest in G0/G1 phase and G2/M phase	Lu et al. (2018)
	GSC	5–7.5	Caused mitochondrial fragmentation and reduced the membrane potential and ATP production	Kuramoto et al. (2017)
Lung cancer	H292	10–80	Overexpression of mir-144–3p induced upregulation of Nrf2 to promote apoptosis	Chen et al. (2018b)
	A549/H460	20–80	Blocked cell cycles progression of the G2/M transition and inducing apoptosis	Qiu et al. (2017)
	A549/H1299	5–20	Activated CHOP pathway	Tang et al. (2016)
	A549/WI-38	10–20	Inhibition of Akt signaling pathway and expression of downstream transcription factor Sp1	Huang et al. (2014)
	MLE-12	10	Reverse lung injury caused by NNK through the mir-144 and MAPK pathways	Li et al. (2020)
Hepatoma	HepG2	10–80	Attenuat p38/JNK/ERK signaling pathway	Chen et al. (2017), Wang et al. (2018)
	HuH7/HepG2	50	Autophagy induced by ULK1/Atg13 and ROS pathways	Niu et al. (2018)
Malignant pleural mesothelioma	MSTO-211H/H28	10–40	Apoptosis was regulated by down-regulating the expression of Sp1	Kim et al. (2015)
Gastric cancer	MKN45/SGC7901/GES-1	15–60	Blocked the Akt/HK2 pathway	Wu et al. (2017)
	BGC-823	20–100	Caused activation of ERK, JNK and p38 MAPK	Hao et al. (2015)
Oral cancer	HSC4/HN22	10–40	Downregulation of Sp1 expression induces apoptotic cell death in OSCC cells	Cho et al. (2014)
	SCC-25	25–100^a^	Decreased the expression of mesenchymal markers N-cadherin	Shen et al. (2014)
Bladder cancer	T24/5637	20–60	Induced ROS-dependent G2/M phases arrest and apoptosis	Jiang et al. (2014), Hong et al. (2019)
Cervical cancer	FaDu	25–125	Induced TRAIL expression was mediated in part by an MAPK signaling pathway involving ERK1/2 and p38	Park et al. (2015)
Lymphoma	T24	20–80	Induce mitochondrial dysfunction, decreased mitochondrial membrane potential	Wang et al. (2015), Yang et al. (2016)

^a^ μg/mL.

In recent years, research into Lico A’s anti-cancer activity has deepened, broadening its scope of anti-cancer effects. Novel advancements have been made in understanding Lico A’s role against oral and nasopharyngeal cancers (Kim et al., 2010; Chuang et al., 2019). Cancer cells’ inherent ability to sustain growth signals and perpetually proliferate underscores the significance of inhibiting their proliferation in cancer treatment. Through an examination of anti-cancer mechanisms and targets, it is evident that Lico A primarily exerts its anti-cancer effect by inducing apoptosis and impeding the cell cycle. The mitochondrial apoptotic pathway is a central conduit through which Lico A induces apoptosis.

## 3 Lico A exerts anti-inflammatory activity

Inflammation is a common and significant pathological response that underlies many conditions, including surface infections and organ-specific ailments such as pneumonia, hepatitis, and nephritis. It involves a delicate balance between proinflammatory factors and the body’s defense mechanisms, which influences the onset, progression, and resolution of inflammation. The NF-κB and Nrf2 pathways play crucial roles in the development of inflammation, and the unique structure of Lico A provides strong anti-inflammatory activity by modulating these pathways.

### 3.1 Lico A achieves anti-inflammatory effects by regulating NF-κB pathway

NF-κB, a nuclear transcription factor widely distributed in various cell types, orchestrates the transcription and expression of genes involved in processes such as cell proliferation, differentiation, and immune response (Maracle et al., 2017; Wang et al., 2018). The NF-κB signaling pathway consists of NF-κB, Inhibitor of NF-κB (IκB), and IκB kinases (IKK). In an inactive state, the NF-κB dimer is bound to IκB. Cellular stimulation leads to IKK activation, promoting the phosphorylation and ubiquitination of IκB, followed by its degradation, freeing the NF-κB dimer to bind to target genes and regulate their expression (Napetschnig and Wu, 2013). Activation of the NF-κB pathway is linked to apoptosis and chronic inflammatory diseases such as rheumatoid arthritis, inflammatory bowel disease, and asthma (Su et al., 2018).

Studies have shown that Lico A can inhibit TNF-α-induced NF-κB transcriptional activity, possibly by suppressing IKK activation and IκB degradation (Tsai et al., 2014). Lico A also inhibits the secretion of IL-1β, IL-6, and TNF-α inflammatory cytokines by down-regulating TLR-4 expression and inhibiting the TLR-4/NF-κB inflammatory signaling pathway (Lv et al., 2019). Moreover, Lico A has shown significant inhibition in LPS-induced microglial cell line BV-2 phosphorylation, suggesting a neuroprotective pharmacological activity (Huang et al., 2017).

### 3.2 Lico A achieves anti-inflammatory effects by regulating Nrf2 pathway

Nrf2, part of the Cap-n-Collar (CNC) regulatory protein family, is a critical transcription factor for cellular antioxidant stress (Moi et al., 1994). Under normal conditions, it remains inactive, bound to Keap1 in the cytoplasm. External stimuli or oxidative stress trigger Nrf2’s dissociation from Keap1, followed by phosphorylation and nuclear transfer. Nrf2 then binds to the antioxidant response element (ARE), initiating the expression of phase II metabolic enzymes and antioxidants, thereby enhancing the body’s resistance to oxidative stress (Tu et al., 2019). The anti-inflammatory impact of the Nrf2 pathway mainly stems from Nrf2 antioxidant pathway activation, which reduces NF-κB’s stress-sensitive expression by lowering IκB phosphorylation and subsequently diminishing inflammation (Chen et al., 2006). Nrf2 and NF-κB pathways mutually inhibit each other (Pedruzzi et al., 2012).

Research has uncovered that Lico A’s anti-arthritis effects depend on the activation of the Keap1-Nrf2 signaling pathway through p62 phosphorylation at the Ser349 site (Su et al., 2018). In the context of neuroinflammation, Lico A protects OGD/R-stimulated rat primary cortical neurons, and counters oxidative stress-induced neuronal damage, and inflammatory reactions by activating the SIRT1/Nrf2 signaling pathway and inhibiting its downstream NF-κB signaling pathway (Liu D. et al., 2018a). Table 2 illustrates the effect of Lico A on different inflammations and the specific mechanisms of action.

**TABLE 2:** The type and mechanism of action of Lico A against inflammation.

Type	Cell model	Animal model	Mechanism	Ref
Asthma	TNF-α and IL-4 induced BEAS-2B	BALB/c mice were sensitized with ovalbumin	Inhibited Th2-associated cytokines	Huang et al. (2019a)
Liver injury	-	LPS/GalN-induced C57BL/6 mice	Inhibition of TLR4-MAPK and NF-κB and Txnip-NLRP3 signaling pathways	Lv et al. (2019)
Mastitis	LPS induced mMEC	LPS perfused BALB/c mice	Inhibited the MAPK and AKT/NF-κB signaling pathways	Guo et al. (2019)
Neuroinflammation	LPS induced RAW 264.7 and BV-2	-	Protect neurons from Aβ- and LPS/IFN-γ-induced toxicity and apoptosis	Lu et al. (2018)
	Primary cultured rat cortical neurons were exposed to OGD/R	-	Counteracts OGD/R-mediated Downregulation of SIRT1, Nrf2 and HO-1, and upregulation of p65	Liu et al. (2018b)
	BV-2 cells stimulated with LPS	Wister rats was given the injection of LPS	Blocked the phosphorylation of ERK1/2 and p65	Huang et al. (2017)
Acne	P. acnes induced primary mouse macrophages and SZ95	P. acnes induces ear swelling in C57BL/6 mice	Inhibited NLRP3 inflammasome	Lee et al. (2018)
Arthritis	RASFs	Collagen-induced arthritis model of DBA/1 mice	Activate of Keap1-Nrf2 signaling	Su et al. (2018)
Acute kidney injury	-	C57BL/6 mice model of LPS-induced AKI	Inhibited LPS-induced NF-κB activation	Hu and Liu (2016)
Ulcerative colitis	-	DSS -induced ulcerative colitis	Downregulation of NF-κB pathway and upregulation of nuclear factor Nrf2 pathway	Liu et al. (2018a)
Skin inflammation	HT1080/HDF	post-shave irritation model	Decreased NF-κB and PGE2 secretion	Sulzberger et al. (2016)

## 4 Other pharmacological activities of Lico A

### 4.1 Improve obesity and lower blood glucose

Obesity, a significant risk factor for chronic diseases including cardiovascular ailments, hypertension, osteoarthritis, specific cancers, and diabetes, is increasingly prevalent worldwide (Tang et al., 2017). It also contributes to nonalcoholic fatty liver disease (NAFLD) and hepatic steatosis (Reccia et al., 2017). Research has shown that Lico A treatment in high-fat diet (HFD)-induced obese mice reduces body weight and decreases inguinal and epididymal adipose tissue compared to HFD-treated mice. Additionally, Lico A improves hepatic steatosis, regulates serum triglycerides, low-density lipoproteins, free fatty acids, and lowers fasting blood glucose levels (Luo et al., 2019). Lico A’s specific lipid-lowering mechanism involves activating the SIRT1/AMPK pathway, reducing fatty acid synthesis, and enhancing lipolysis and beta-oxidation in hepatocytes (Liou et al., 2019).

Inducing the browning of white adipose tissue (WAT) represents a promising strategy for obesity treatment (Kajimura et al., 2010; Bartelt et al., 2011). Lico A enhances the expression of brown fat markers, reducing obesity and restoring metabolic equilibrium (Lee et al., 2018).

### 4.2 Anti-bacterial and fungal effects

Salmonellosis, caused by multi-drug-resistant *Salmonella Typhimurium*, poses a global public health threat (Behravesh et al., 2014). Lico A inhibits the growth of *S. Typhimurium* at MIC levels of 62.5–1,000 μg/mL, with an MBC value > 1,000 μg/mL (Hosseinzadeh et al., 2018). Additionally, Lico A exhibits substantial antifungal activity against *Candida* albicans, inhibiting biofilm formation by 35%–60%, and suppressing yeast-hyphal transformation and protease secretion (Seleem et al., 2016).

### 4.3 Antiparasitic effect

Toxoplasma gondii, the causative agent of toxoplasmosis, poses significant public health challenges (Ajzenberg et al., 2016). Lico A effectively inhibits T. gondii proliferation in a dose- and time-dependent manner with low cytotoxicity against HFF host cells (Si et al., 2018). Additionally, Lico A reduces the total number of Schistosoma mansoni eggs, likely by increasing ROS production and inducing the death of adult Schistosoma mansoni (Souza et al., 2017).

### 4.4 Strengthen bone formation and increase bone mass

Osteoporosis, characterized by loss of bone microstructure, heightens fracture risk (Smith and Walker, 1976). The role of bone marrow mesenchymal stem cells (BMSCs) in osteoporosis has drawn increasing attention. Lico A exerts a potent influence on BMSC osteogenic differentiation and mineralization by up-regulating FasL, and further modulating ERK and GSK-3β-catenin. Through the activation of intraosseous bone formation and partial inhibition of bone resorption in an acute estrogen deficiency model, Lico A administration restores or protects bone mass in disease states (Ming et al., 2015).

### 4.5 Intestinal protective activity

In a recent study, the intestinal protective effect of Lico A was revealed. It was indicated that Lico A could promote intestinal epithelial renewal to exert intestinal protective effect. The mechanism involves regulating T-UCRs (transcripts from ultra-conserved regions) (Wang et al., 2024).

## 5 Discussion

Anti-cancer and anti-inflammatory properties are the main characteristic bioactivities of Lico A, compared with other pharmacological activities. It has been reported that there is a close relationship between inflammation and cancer. On one hand, the persistent inflammatory microenvironment instigates tumors by initiating specific genetic mutations (Botta et al., 2016). On the other, a growing body of evidence indicates that tumor-related inflammation promotes angiogenesis and metastasis. This loop regulation suggests that Lico A has great potential in cancer prevention for its action of mechanism. The NF-κB pathway, recognized as a classical inflammation pathway, is a key channel through which Lico A exerts its effects against inflammation conditions such as hepatitis, neuroinflammation, and mastitis (Sen and Baltimore, 1986). The MAPK pathway, implicated in both inflammation and cancer, is another target of Lico A. By influencing these targets, Lico A delivers either anti-inflammatory or anti-cancer effects.

In addition to anti-inflammation and anti-cancer activities, Lico A can also elicit other activities like Anti-bacterial and fungal, Antiparasitic, and intestinal protective effects. However, investigations on these bioactivities are relatively few and lack systematic in-depth studies to fully demonstrate the potential of Lico A, which hindered the further development as a natural bioactive molecule and becomes the key limitation for current research of Lico A.

In addition to inflammation and cancer, recent studies showed the modulatory effect of Lico A on Post-transcriptional regulation. Post-transcriptional regulation refers to the regulation of gene expression after RNA transcription and is a characteristic of gene expression in eukaryotes (Dykes and Emanueli, 2017). The initial transcript must undergo a series of processes before transforming into a functional mature mRNA, serving as a template for protein translation (Masamha and Wagner, 2017). Various mechanisms regulate and control the type and quantity of gene expression during this process. Current research focuses on non-coding RNA (ncRNA) such as miRNA, lncRNA, and circRNA (Tezcan et al., 2019). It is concluded that Lico A can regulate the Nrf2 and MAPK pathways by modulating miR-144, indicating that Lico A has the potential to regulate ncRNA, providing new avenues for studying its pharmacological mechanisms. Moreover, a recent study shows that Lico A can modulate T-UCR regulation. As T-UCRs are also non-coding RNAs and have good conservative characteristics among rats, mice, and humans, playing a fundamental and primary role in gene regulation, more research should be performed to explore the effect of Lico A on posttranscriptional gene regulation.
